# Physiological regulation of calcium and phosphorus utilization in laying hens

**DOI:** 10.3389/fphys.2023.1112499

**Published:** 2023-02-07

**Authors:** Micaela Sinclair-Black, R. Alejandra Garcia, Laura E. Ellestad

**Affiliations:** Department of Poultry Science, University of Georgia, Athens, GA, United States

**Keywords:** laying hen, calcium, phosphorus, vitamin D_3_, skeletal health, egg formation

## Abstract

Commercial laying hens can produce one egg approximately every 24 h. During this process, regulatory systems that control vitamin D_3_ metabolism, calcium and phosphorus homeostasis, and intestinal uptake of these minerals work in concert to deliver components required for eggshell calcification and bone mineralization. Commercial production cycles have been extended in recent years to last through 100 weeks of age, and older hens often exhibit an increased prevalence of skeletal fractures and poor eggshell quality. Issues such as these arise, in part, through imbalances that occur in calcium and phosphorus utilization as hens age. As a result, an in-depth understanding of the mechanisms that drive calcium and phosphorus uptake and utilization is required to develop solutions to these welfare and economic challenges. This paper reviews factors that influence calcium and phosphorus homeostasis in laying hens, including eggshell formation and development and roles of cortical and medullary bone. Metabolism and actions of vitamin D_3_ and physiological regulation of calcium and phosphorus homeostasis in key tissues are also discussed. Areas that require further research in avian species, such as the role of fibroblast growth factor 23 in these processes and the metabolism and action of bioactive vitamin D_3_, are highlighted and the importance of using emerging technologies and establishing *in vitro* systems to perform functional and mechanistic studies is emphasized.

## 1 Introduction

As the global population grows, there is increased demand for affordable, high-quality, and sustainable protein sources like table eggs. Commercial laying hens have been selected to increase eggs produced per hen lifetime, with production cycles now lasting past 100 weeks of age. Economic and sustainability benefits of extended lay persistency include decreased cost and environmental impact on a per-egg basis (Bain et al., 2016), but there are challenges associated with egg quality and bird welfare as hens age.

Older hens often produce larger, weak-shelled eggs (Al-Batshan et al., 1994) and exhibit compromised skeletal structure. Efficiency of intestinal calcium absorption decreases with age (Diana et al., 2021), leading to increased reliance on bone-derived calcium contributing to fractures (Gregory and Wilkins, 1989). Elucidating physiological mechanisms responsible for the uptake and utilization of calcium and phosphorus throughout the hen’s productive lifecycle will provide insights that can be used to develop strategies limiting economic losses to producers and improving animal welfare.

## 2 Egg formation

Commercial laying hens produce an egg approximately every 24 h (Nys and Guyot, 2011) and must efficiently regulate calcium and phosphorus utilization for eggshell calcification and cuticle formation, respectively (Cusack et al., 2003). Ovulation occurs 15–75 min after oviposition, or egg-laying (Sturkie and Mueller, 1976), and the follicle resides in the infundibulum for under 30 min (Sah and Mishra, 2018). It continues into the magnum where albumen is added over the next 3.25–3.5 h (Nys and Guyot, 2011) and enters the isthmus where inner and outer shell membranes are deposited around the albumen (Warren and Scott, 1935). Organic eggshell matrix proteins (e.g. ovalbumins, osteopontins, ovocleidins, ovocalyxins) and calcium carbonate are deposited onto the outer shell membrane (Hincke et al., 2010) in the shell gland, and the eggshell forms over the final 19–20 h (Nys and Guyot, 2011; Gautron et al., 2021).

As previously described (Nys and Guyot, 2011; Gautron et al., 2021), the eggshell develops as distinct mamillary, palisade, and cuticle layers deposited from interior to exterior. During mineralization of the mammillary and palisade layers, deposition of amorphous calcium carbonate is followed by its transformation into calcite crystals (Rodriguez-Navarro et al., 2015). Initially, the mamillary layer forms at nucleation sites laid on the outer shell membrane between 5–6 h post-oviposition (HPOP) and the mamillary core develops between 7–10 HPOP. Large calcite crystal units form the columnar palisade layer between 10–22 HPOP, and the cuticle forms an organic film preventing bacterial penetration of the egg about 2 h before oviposition. A calcium and phosphorus-rich hydroxyapatite [Ca_10_(PO_4_)_6_(OH)_2_] crystal layer lies just internal to the cuticle (Wedral et al., 1974; Cusack et al., 2003). Since phosphorus is a potent inhibitor of calcite formation (Bachra et al., 1963; Simkiss, 1964), some authors speculate that these crystals (Dennis et al., 1996) or the secretion of phosphate-containing organic eggshell constituents towards the end of shell formation (Nys et al., 1991) may be involved in terminating calcification.

## 3 Bone development and remodeling

Since most eggshell calcification takes place in the dark when dietary calcium is largely unavailable, hens mobilize approximately 20%–40% of calcium required for eggshell formation from bone (Comar and Driggers, 1949). Structural cortical and trabecular bone with highly organized hydroxyapatite crystals is formed during embryonic and juvenile development. After structural bone deposition subsides (Hudson et al., 1993), increased circulating estrogen at the onset of sexual maturity around 18 weeks of age leads to development of medullary bone in pneumatic and long bones (Whitehead, 2004). Medullary bone is highly vascularized with randomly orientated hydroxyapatite crystals (Dacke et al., 1993), allowing for rapid anabolism and catabolism of hydroxyapatite during egg formation. Since hydroxyapatite is composed of calcium and phosphorus, bone resorption releases both minerals into circulation as ionized calcium (iCa^2+^) and inorganic phosphate [PO_4_^3−^ (P_i_)] that must be utilized for shell formation or excreted.

Medullary bone undergoes remineralization when eggshell calcification is not occurring (Wilson and Duff, 1990; Kerschnitzki et al., 2014) and is resorbed during eggshell calcification (Van de Velde et al., 1984b) through increased osteoclast activity driven by parathyroid hormone (PTH) and the bioactive form of vitamin D_3_, 1,25-dihydroxycholecalciferol [1,25(OH)_2_D_3_] (Taylor and Belanger, 1969). When PTH binds PTH receptor 1 (PTH1R) on osteocytes (Silve et al., 1982; Zhao et al., 2002), receptor activator of nuclear factor-kappa B ligand (RANKL) is secreted and interacts with receptor activator of nuclear factor-kappa B (RANK) on osteoclasts, stimulating bone resorption. Additionally, PTH increases osteoclast vacuolar-type adenosine triphosphatase (V-ATPase) activity, causing intracellular acidification required for bone breakdown (Liu et al., 2016). Osteoclast activity increases nine-fold during shell calcification (Van de Velde et al., 1984b), and osteoporosis can develop when osteoclasts resorb structural bone once medullary bone is depleted. Dysregulation of medullary bone remodeling may contribute to development of osteoporosis in aged hens, which exhibit increased medullary bone expression of the resorption marker carbonic anhydrase 2 (*CA2*) and vitamin D_3_ receptor (*VDR*), as well as reduced expression of accretion proteins like collagen type 1 alpha 1 (*COL1A1*), relative to younger hens (Gloux et al., 2020b).

## 4 Vitamin D_3_ metabolism and mechanism of action

Skeletal integrity and eggshell quality depend on 1,25(OH)_2_D_3_ because of its role in regulating calcium and phosphorus homeostasis. Dietary vitamin D_3_ is constitutively hydroxylated in the liver by a 25-hydroxylase enzyme encoded by the *CYP2R1* gene (Watanabe et al., 2013), with >90% converted into 25(OH)D_3_ (Heaney et al., 2008; San Martin Diaz, 2018). A second, more tightly regulated hydroxylation occurs in the kidney at the 1α-carbon to form 1,25(OH)_2_D_3_ (Jones et al., 1998). In mammals and fish, this is carried out by an enzyme encoded by *CYP27B1* (Monkawa et al., 1997; Shinki et al., 1997; Chun et al., 2014); however, this gene has not been identified in chickens and the enzyme responsible is currently unknown despite recent publications that have reported measuring expression of *CYP27B1* mRNA or an equivalent (Shanmugasundaram and Selvaraj, 2012; Gloux et al., 2020a; Gloux et al., 2020b; Yan et al., 2022). Investigation into transcripts amplified reveals these are an enzyme involved in retinoic acid metabolism (*CYP27C1*) or one identified as vitamin D_3_ hydroxylase-associated protein (Ettinger et al., 1994; Ettinger and DeLuca, 1995), neither of which have demonstrable 1α-hydroxylase activity. PTH stimulates 1α-hydroxylation of vitamin D_3_ when circulating iCa^2+^ and 1,25(OH)_2_D_3_ are low; however, the efficiency of this may decrease with age (Abe et al., 1982; Gloux et al., 2020b). During periods of elevated circulating 1,25(OH)_2_D_3_, 1α-hydroxylase is inhibited and 24-hydroxylase, encoded for by *CYP24A1*, is upregulated. The 24-hydroxylase enzyme inactivates 25(OH)D_3_ by producing biologically inert 24,25(OH)_2_D_3_ or 1,24,25(OH)_3_D_3_ (Holick et al., 1973; Omdahl et al., 2002), thereby preventing excessive bone resorption and intestinal calcium absorption. Hydroxylation of 25(OH)D_3_ into either active or inactive metabolites provides an additional level of control by fine-tuning the availability of this hormone.

Vitamin D_3_ affects calcium and phosphorus homeostasis through its influence on expression and activity of transport and chaperone molecules for these minerals. When bound by 1,25(OH)_2_D_3_, VDR acts as a ligand-activated transcription factor that enters the nucleus to form a heterodimeric complex with retinoid-X-receptor alpha (RXRA) or gamma (RXRG) and binds vitamin D_3_ response elements (VDRE) in regulatory regions of vitamin D_3_-responsive genes (Bikle, 2014). Not all tissues respond to 1,25(OH)_2_D_3_ in the same way. For example, shell gland calbindin D-28k (*CALB1*) expression does not appear to be influenced by 1,25(OH)_2_D_3_ (Bar et al., 1977), unlike that in the kidney and small intestine (Taylor and Wasserman, 1972). It may be under the control of estrogen (Nys et al., 1992; Corradino et al., 1993), driven by half-palindromic estrogen response elements in the *CALB1* promoter as has been shown in mice (Gill and Christakos, 1995), and intracellular calcium levels (Corradino, 1993). Since CALB1 in shell gland, intestine, and kidney share the same electrophoretic mobility, amino acid composition, and immunoreactivity, it is likely the same protein (Fullmer et al., 1976); however, estrogen receptor rather than VDR could be a key regulatory protein driving its expression in the shell gland.

## 5 Calcium homeostasis and transport

Regulation of calcium homeostasis is required to maintain the daily flux of this mineral in laying hens. The highest demand occurs when the eggshell is actively calcifying during the nocturnal fast, and hens must rely on reduced intestinal pH to solubilize coarse limestone retained in the gizzard (Scanes et al., 1987). This occurs through stimulation of H^+^/K^+^-ATPase activity in the proventriculus (Guinotte et al., 1995) and subsequent secretion of hydrochloric acid (Guinotte et al., 1993).

During eggshell calcification, decreased circulating iCa^2+^ due to high demand by the shell gland (Parsons and Combs, 1980) is detected by calcium-sensing receptor (CASR) (Hofer and Brown, 2003) and leads to PTH secretion from the parathyroid gland (Van de Velde et al., 1984a; Singh et al., 1986). PTH rectifies circulating iCa^2+^ back to its homeostatic range by stimulating bone resorption (Taylor and Belanger, 1969) and increasing 1,25(OH)D_3_ production in the kidney (Brenza and DeLuca, 2000); 1,25(OH)D_3_ works to increase calcium absorption from the small intestine (Spencer et al., 1978; Chandra et al., 1990) and reabsorption in the kidney (Jande et al., 1981).

Calcitonin (CALC), produced within ultimobranchial bodies near the thyroid gland (Copp et al., 1967; Kraintz and Intscher, 1969), may reduce iCa^2+^ in chickens (Luck et al., 1979), and expression of CALC receptor (*CALCR*) in shell gland, kidney, and bone of laying hens (Yasuoka et al., 1998; Ieda et al., 2001) suggests it could play a role in regulating calcium homeostasis. However, unlike in mammals, CALC does not influence avian osteoclast activity under normal physiological conditions (Nicholson et al., 1987; Eliam et al., 1988), nor does it appear to affect renal cyclic adenosine monophosphate formation in chickens or pigeons (Dousa, 1974). This implies that avian CALCR could use alternative intracellular signaling pathways or that CALC does not have the same effect on bone as it does in mammals. At present, there is limited evidence that CALC strongly influences calcium homeostasis in birds, suggesting it may not be an important regulator of calcium availability for egg production.

Calcium absorption from the small intestine appears to fluctuate throughout the daily egg formation cycle (Hurwitz and Bar, 1969; Hurwitz et al., 1973) and is thought to occur primarily in the duodenum and jejunum, with smaller amounts absorbed in the ileum (Hurwitz and Bar, 1965; Hurwitz and Bar, 1968). Intestinal calcium uptake occurs through active transcellular and passive paracellular pathways. Active transcellular absorption accounts for most calcium uptake and involves ATPase plasma membrane calcium transporting 1 (ATP2B1), 2 (ATP2B2), and 4 (ATP2B4), sodium-calcium exchanger 1 (NCX1), transient receptor potential cation channel subfamilies C member 1 (TRPC1), M member 7 (TRPM7), and V member 2 (TRPV2), and CALB1 (Bar, 2009; Gloux et al., 2019). Passive paracellular calcium absorption likely takes place *via* tight junction proteins 1 (TJP1), 2 (TJP2), and 3 (TJP3), claudin 2 (CLDN2) and 12 (CLDN12), and occludin (OCLN) (Gloux et al., 2019; Gloux et al., 2020b). Findings suggest that intestinal capacity for calcium absorption could change with age, as expression of some transcellular (*ATP2B4*, *TRPV2*) and paracellular (*TJP3*, *CLDN2*, *OCLN*) transporters decreased in older hens (Gloux et al., 2020b). Calcium transport in the shell gland (Brionne et al., 2014) and kidney occurs through many of these same proteins, with the addition of transient receptor potential cation channel subfamily V member 6 (TRPV6) in the kidney (Proszkowiec-Weglarz and Angel, 2013; Gautron et al., 2021; Wang et al., 2022). This has been shown to decrease with age in hens (Gloux et al., 2020b), indicating that the calcium-handling capacity of the kidney is perturbed in older layers. In addition to the above-listed transporters, recent findings suggest vesicular transport systems may export calcium into the shell gland lumen (Stapane et al., 2020).

## 6 Phosphorus homeostasis and transport

Approximately 80% of phosphorus is stored in the skeleton as hydroxyapatite. It is released when bone is resorbed during eggshell calcification, and this excess P_i_ (Nys et al., 1986; Frost and Roland, 1990) must be excreted to negate toxic effects. Maintenance of circulating P_i_ occurs in the kidney, small intestine, and bone (Michigami et al., 2018) and is primarily regulated by fibroblast growth factor 23 (FGF23); however, PTH and 1,25(OH)_2_D_3_ also influence it through their actions on calcium homeostasis (Ren et al., 2020).

In mice (Perwad et al., 2005) and laying hens (Ren et al., 2017; Wang et al., 2018; Gloux et al., 2020a; Ren et al., 2020), hyperphosphatemia increases FGF23 production in bone. It has been shown to bind to one of four FGF receptors (FGFR1-4) along with the co-receptor klotho (KL) in mammals (Razzaque, 2009), and this complex induces expression of P_i_ transport proteins that mediate FGF23’s phosphaturic effects. Laying hens express *FGF23* mRNA in both medullary and structural bone (Hadley et al., 2016; Wang et al., 2018), and increases in its expression occur as they age (Gloux et al., 2020b). Furthermore, hens exhibit *FGFR1-4* and *KL* mRNA expression in the kidney, intestine, and bones (Ren et al., 2020). Immunoneutralization of FGF23 in laying hens led to increased plasma P_i_ and bone ash under phosphorus-deficient conditions (Bobeck et al., 2012; Ren et al., 2017), and limiting dietary P_i_ in laying hens reduced circulating P_i_, suppressed bone *FGF23* mRNA, circulating FGF23, and renal sodium-dependent P_i_ transporter IIa (*NaP*_*i*_ *IIa*) expression, and induced duodenal sodium-dependent P_i_ transporter IIb (*NaP*_*i*_ *IIb*) expression (Ren et al., 2020). These changes corresponded with reduced phosphorus excretion and increased calcium excretion. Studies conducted in mammals have found that FGF23 directly inhibited PTH secretion (Ben-Dov et al., 2007), decreased renal P_i_ transporter 2 (*P*_*i*_ *T-*2) expression (Tomoe et al., 2009), and limited 1,25(OH)_2_D_3_ production in the kidney, in part through upregulation of 24-hydroxylase (Perwad et al., 2007). In hens, similarities exist whereby elevated medullary *FGF23* mRNA during eggshell calcification was followed by increased renal mRNA for *CYP24A1* after oviposition, which may have led to observed reductions in 1,25(OH)_2_D_3_ (Gloux et al., 2020a).

In birds, 1,25(OH)_2_D_3_ appears to directly stimulate renal P_i_ reabsorption in the short-term and inhibit it in the long-term (Liang et al., 1982; Liang et al., 1984). Renal P_i_ reabsorption was decreased, and therefore P_i_ excretion increased, by PTH (Wideman and Braun, 1981). The capacity of the kidney to regulate P_i_ balance could change with age, as expression of *NaP*_*i*_ *IIa* and P_i_ transporter 1 (*P*_*i*_ *T-1*) in kidney decreased in older hens (Gloux et al., 2020b). Since PTH stimulates production of 1,25(OH)_2_D_3_, it indirectly increases P_i_ absorption from the intestine (Liao et al., 2017). Intestinal P_i_ uptake in chickens is thought to be mediated by P_i_T-1, P_i_T-2, NaP_i_IIa, and NaP_i_IIb (Yan et al., 2007; Huber et al., 2015; Li et al., 2018), with NaP_i_IIb as the primary transporter in the duodenum and jejunum and P_i_T-1 as the primary transporter in the ileum (Gloux et al., 2019).

## 7 Discussion

**FIGURE 1:**
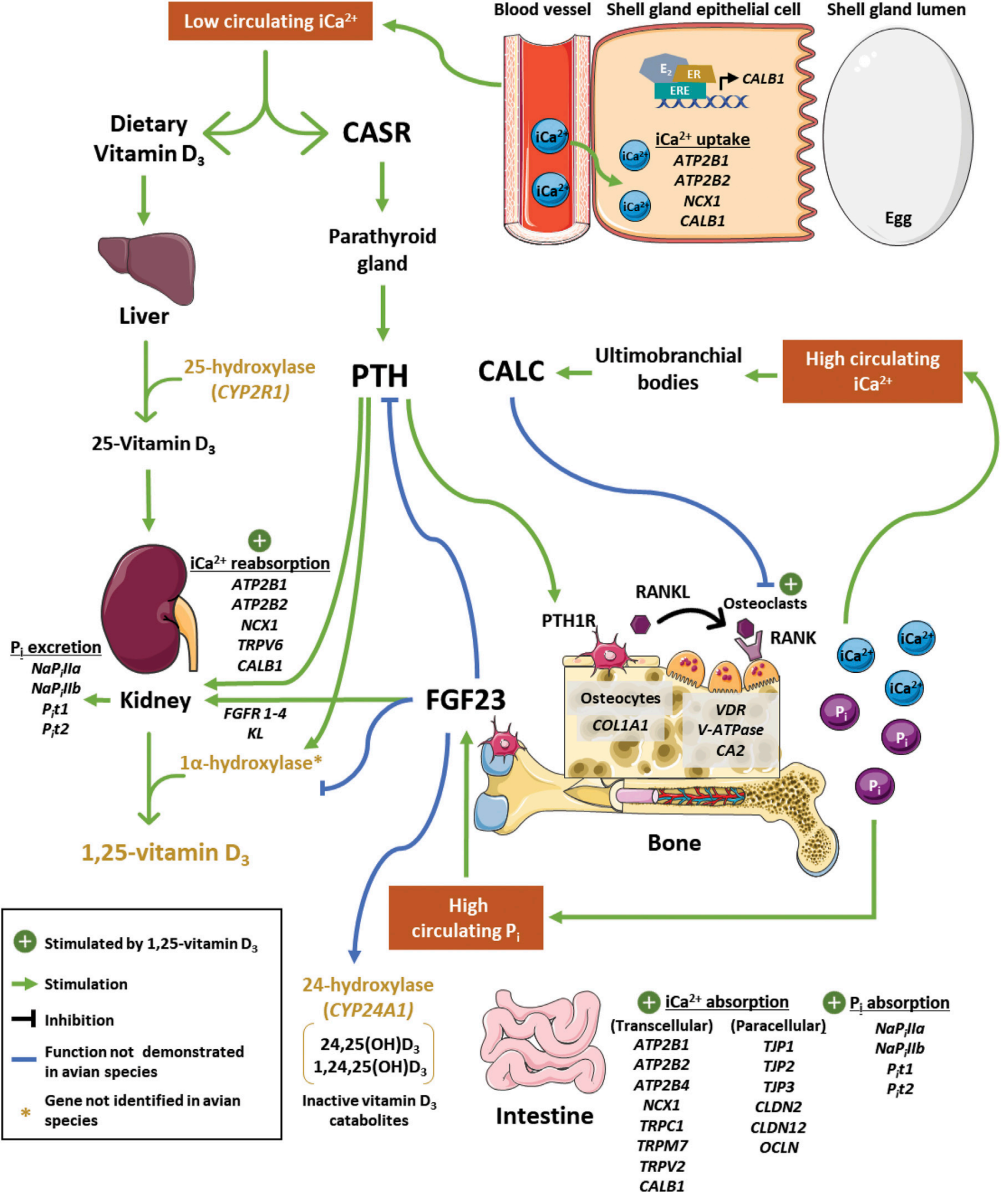
Regulation of calcium and phosphorus homeostasis during eggshell mineralization in laying hens. During eggshell calcification, high demand for calcium decreases circulating ionized calcium (iCa^2+^). Low iCa^2+^ is detected by calcium-sensing receptor (CASR), which stimulates parathyroid hormone (PTH) secretion from the parathyroid gland. Secreted PTH binds to PTH receptor 1 (PTH1R) on osteocytes to promote interaction between receptor activator of nuclear factor-kappa B (RANK) and RANK ligand (RANKL) on the osteoclast surface. This induces vacuolar-type adenosine triphosphatase (V-ATPase) production to facilitate bone resorption alongside carbonic anhydrase 2 (CA2). In contrast, bone accretion is facilitated by deposition of matrix proteins such as collagen type 1 alpha 1 (COL1A1). In the kidney, PTH stimulates inorganic phosphate (P_i_) excretion and upregulates production of 1,25(OH)_2_D_3_. Bioactive 1,25(OH)_2_D_3_, which binds to vitamin D_3_ receptor (VDR), stimulates osteoclast activity, calcium transport in the kidney, and calcium and phosphorus uptake in the intestine. Impacts of 1,25(OH)_2_D_3_ in the shell gland and on paracellular intestinal calcium uptake still need to be elucidated. Transcellular transport of calcium in these tissues is thought to occur through ATPase plasma membrane calcium transporting 1, 2, and 4 (ATP2B1, ATB2B2, ATP2B4; intestine only), sodium-calcium exchanger 1 (NCX1), calbindin-28K (CALB1), transient receptor potential cation channels subfamily C member 1 (TRPC1; intestine only), transient receptor potential cation channels subfamily M member 7 (TRPM7; intestine only), and transient receptor potential cation channel subfamily V member two and six (TRPV2, intestine only; TRPV6, kidney only). Paracellular transport in the intestine is achieved by tight junction proteins 1, 2, and 3 (TJP1, TJP2, TJP3), claudin 2 and 12 (CLDN2, CLDN12) and occludin (OCLN). Transport of phosphorus in these tissues is thought to occur by sodium-dependent phosphorus transporters IIa and IIb (NaP_i_IIa and NaP_i_IIb) and sodium-dependent inorganic phosphorus transporters 1 and 2 (P_i_t1 and P_i_t2). Shell gland calcium transport by CALB1 may be under the control of estradiol (E_2_) through estrogen receptor (ER) interaction with estrogen-response elements (EREs) in its promoter region. Bone breakdown releases P_i_ into circulation, which induces production of fibroblast growth factor 23 (FGF23). In chickens and mammals, this peptide stimulates renal phosphorus excretion, which has been shown to be mediated through its binding to FGF23 receptors (FGFR1, FGFR2, FGFR3, FGFR4) and co-receptor klotho (KL) in mammals. In mice, FGF23 has also been shown to exhibit negative feedback on PTH and 1α-hydroxylase activity, as well as stimulate 24-hydroxylase activity. During periods of elevated iCa^2+^, calcitonin (CALC) is secreted from cells in ultimobranchial bodies to inhibit osteoclast activity in mammals, but its effects in birds are unclear. Further investigation into several of these processes and how transporters function in a tissue-specific manner is required to determine their role in calcium and phosphorus homeostasis in chickens. Parts of the figure were drawn by using pictures from servier medical art, licensed under a creative commons attribution 3.0 unported license (https://creativecommons.org/licenses/by/3.0/).

This review investigates physiological mechanisms influencing calcium and phosphorus utilization in laying hens during egg production (Figure 1). Age-dependent changes in levels of FGF23, 1,25(OH)_2_D_3_, and several calcium and phosphorus transporters in the intestine and kidney suggest that the ability of hens to maintain adequate mineral balance for optimal shell strength and bone health is compromised during extended lay. This leads to deterioration of structural bone when the rate of medullary bone resorption required for eggshell calcification exceeds that of remineralization during periods outside eggshell development, predisposing hens to fractures that negatively impact their welfare and reduce egg production in an age-dependent fashion (Rufener et al., 2019). To maintain healthy, high-producing hens throughout extended production, skeletal development should be prioritized during rearing to ensure adequate deposition of structural bone prior to initiation of medullary bone accretion.

Improvements in laying hen skeletal health require an in-depth understanding of regulatory systems driving calcium and phosphorus utilization and how they change with age. Further research on how FGF23 influences PTH secretion, vitamin D_3_ metabolism, and other aspects of calcium and phosphorus homeostasis in birds is necessary. Though a role for FGF23 in regulating P_i_ homeostasis in layers has been supported by the findings described above, functional and mechanistic studies demonstrating its direct involvement are limited. As there are differences in medullary bone expression of *FGF23* mRNA with age (Gloux et al., 2020b), and FGF23 appears to influence phosphorus and calcium balance (Bobeck et al., 2012; Ren et al., 2017; Ren et al., 2020), understanding effects of FGF23 on mineral homeostasis and how to manage changes across the production cycle is crucial for maintaining skeletal health and egg production throughout extended lay.

A second area needing further elucidation is the metabolism and action of vitamin D_3_. The gene encoding 1-α hydroxylase has not been identified in avian species, hindering mechanistic studies of its activity. Characterization of *CYP27B1* or a functional equivalent would provide valuable insights into ways that vitamin D_3_ metabolism could be harnessed to improve eggshell integrity and skeletal welfare in layers, including using selection strategies for hens that exhibit stronger bones and eggshells. Furthermore, the influence of 1,25(OH)_2_D_3_ on shell gland calcium transport has been questioned due to unresponsiveness of typical 1,25(OH)_2_D_3_-dependent proteins (Bar et al., 1977; Bar, 2008); additional studies are needed to confirm if this applies to other aspects of shell gland calcium transport. This is especially important, as regulation of ionic calcium transfer into the shell gland lumen is poorly understood (Nys et al., 2022) despite it being a limiting factor in calcium supply to the eggshell (Cohen et al., 1978), so alterations in this process with age likely contribute to decreased shell quality in older hens.

Though a better picture of laying hen calcium, phosphorus, and vitamin D_3_ metabolism has emerged in recent years, critical knowledge gaps exist and much of our understanding of these homeostatic mechanisms is derived from mammalian research. However, hens undergo additional biological processes such as development and maintenance of medullary bone and eggshell calcification, so direct inferences from mammals to birds may be flawed. Availability of the chicken genome in conjunction with “omics” approaches should help identify relevant gene networks across tissues that are involved in these processes, allowing development of testable hypotheses that can be used to discern functionality where it is lacking. Establishment of reliable *in vitro* models for bone, kidney, and shell gland and validated assays for functional proteins would greatly facilitate fundamental, mechanistic studies on these systems. This is essential for generating successful nutritional and genetic management strategies that prioritize skeletal welfare throughout the productive lifecycle of the hen.
